# Differentially Expressed miRNAs Influence Metabolic Processes in Pituitary Oncocytoma

**DOI:** 10.1007/s11064-019-02789-2

**Published:** 2019-04-03

**Authors:** Lilla Krokker, Gábor Nyírő, Lilla Reiniger, Ottó Darvasi, Nikolette Szücs, Sándor Czirják, Miklós Tóth, Péter Igaz, Attila Patócs, Henriett Butz

**Affiliations:** 1 Momentum Hereditary Endocrine Tumours Research Group, Semmelweis University, 46 Szentkiralyi Street, Budapest, 1088 Hungary; 2 2nd Department of Internal Medicine, Semmelweis University, Budapest, Hungary; 3 MTA-SE Molecular Medicine Research Group, Hungarian Academy of Sciences and Semmelweis University, Budapest, Hungary; 4 1st Department of Pathology and Experimental Cancer Research, Semmelweis University, Budapest, Hungary; 5 National Institute of Clinical Neurosciences, Budapest, Hungary; 6 Department of Laboratory Medicine, Semmelweis University, Budapest, Hungary

**Keywords:** Pituitary adenoma, Oncocytoma, miRNA, Biomarker, Next generation sequencing

## Abstract

Spindle cell oncocytomas (SCO) of the pituitary are rare tumors accounting for 0.1–0.4% of all sellar tumors. Due to their rarity, little information is available regarding their pathogenesis. Our aim was to investigate miRNA expression profile of pituitary oncocytomas. Total RNA was extracted from 9 formalin-fixed paraffin embedded pituitary samples (4 primary, 3 recurrent oncocytomas and 2 normal tissues). Next-generation sequencing was performed for miRNA profiling. Transcriptome data of additional 6 samples’ were obtained from NBCI GEO database for gene expression reanalysis and tissue-specific target prediction. Bioinformatical analysis, in vitro miRNA mimics transfection, luciferase reporter system and AlamarBlue assay were applied to characterize miRNA’s function. 54 differentially expressed miRNAs and 485 genes in pituitary SCO vs. normal tissue and 8 miRNAs in recurrent vs. primary SCO were determined. Global miRNA downregulation and decreased level of DROSHA were detected in SCO samples vs. normal tissue. Transcriptome analysis revealed cell cycle alterations while miRNAs influenced mainly metabolic processes (tricarboxylic acid cycle-TCA, carbohydrate, lipid metabolism). Through miRNA-target interaction network the overexpressed Aconitase 2 potentially targeted by two downregulated miRNAs (miR-744-5p, miR-127-3p) was revealed. ACO2 and miR-744-5p interaction was validated by luciferase assay. MiR-127-3p and miR-744-5p significantly decreased cell proliferation in vitro. Our study firstly reported miRNA profile of pituitary oncocytoma. Our results suggest that tumor suppressor miRNAs may have an essential role in the pathogenesis of pituitary oncocytoma. Earlier reports showed downregulated TCA cycle in SCO which is extended by our results adding the role of miR-744-5p targeting ACO2.

**Electronic supplementary material:**

The online version of this article (10.1007/s11064-019-02789-2) contains supplementary material, which is available to authorized users.

## Introduction

Prevalence rate of primary tumors of the anterior pituitary varies 10–22% of all intracranial tumors, however, clinically relevant pituitary adenomas appear more rarely [1, 2]. Adenomas of the anterior pituitary are the most common tumor type in the sellar region followed by craniopharyngioma, meningioma and posterior pituitary tumors (PPT) [3]. Based on the WHO 2017 classification, the main tumors of neurohypophysis are pituicytomas, granular cell tumors, spindle cell oncocytomas (SCO) and sellar ependymomas. According to the current hypothesis, PPTs originate from specific morphological variants of pituicytes (specialized glial cells, i.e. pituicytomas from the light/major variant, granular cell tumors from the granular variant, spindle cell oncocytomas from the oncocytic variant and sellar ependymomas from the ependymal pituicytes) [4]. PPTs show a common immunohistochemical feature, diffuse nuclear expression for the thyroid transcription factor-1 (TTF-1), a homeobox transcription factor [4]. Spindle cell oncocytomas (SCO or oncocytomas) are rare, benign tumors accounting for 0.1–0.4% of all sellar tumors [5]. Until mid-2017, there have been only 34 cases published [3]. Although some hormonally active oncocytomas have been described [6, 7], PPTs are low-grade, non-neuroendocrine neoplasms clinically presenting with symptoms related to mass effects [4, 8, 9].

Due to its rarity, little information is available regarding the pathogenesis of SCO. Exome sequencing confirmed low genomic mutation rate and a neutral copy-number profile [10]. Enhanced MAPK activity was confirmed by strong phospho-ERK staining (n = 4) [10]. In addition, SCOs are characterized by accumulation of mitochondria in the cytoplasm revealed by ultrastructure analysis [11]. Indeed, mitochondrial Complex I disruptive variants in pituitary oncocytomas were described as somatic modifiers of tumorigenesis most likely contributing not only to the development of oncocytic change but also to a less aggressive tumor phenotype [12]. However Complex I mutations lead to dysfunctional respiratory function, Carelli et al. described that Complex I mutations themselves were not sufficient for mitochondrial proliferation [13].

Proteomic analysis showed that 14-3-3η was overexpressed in oncocytomas, and inhibited lactate dehydrogenase A (LDHA) through direct interaction [14]. In line with this, the authors detected disrupted glycolysis and decreased level of lactate together with reduced expression of lactate dehydrogenase A (LDHA) in pituitary oncocytoma tissue specimens by metabolic analysis (NanoLC-MS/MS) [14]. However, hypoxia-response signaling pathway was not upregulated in pituitary oncocytomas, thereby failing to enhance glycolysis [14]. Thus, 14-3-3η was capable of inhibiting glycolysis, which together with dysfunctional mitochondria resulted in an increased mitochondrial biogenesis [14].

MicroRNAs (miRNAs) are small regulatory RNAs that have an important role in elementary biological processes, such as cell proliferation, cell differentiation and cell death. Correlation between miRNAs and cancer and abnormal expression of miRNAs have been shown in a variety of tumor tissues including pituitary adenomas [14–17]. By regulating their target genes expression, miRNAs can play either oncogenic or tumor suppressor functions [17].

Herein, for the first time, we investigated miRNA expression profile of pituitary oncocytomas and evaluated the role of differentially expressed miRNAs by complex bioinformatical approaches.

## Methods

### Patients and Samples

Nine formalin fixed paraffin embedded pituitary samples (4 primary oncocytomas, 3 recurrent oncocytomas and 2 normal tissues) from the archive of the 1st Department of Pathology and Experimental Cancer Research, Semmelweis University, Budapest, Hungary were studied (Table 1). The adjacent non-tumorous tissue of 2 pituitary oncocytoma cases were macrodissected and used as normal control. Pituitary oncocytomas were surgically removed at the National Institute of Clinical Neurosciences between 2009 and 2018. The study was approved by the Scientific and Research Committee of the Medical Research Council of Hungary (0618/15).

**Table 1:** Patient and sample characteristics

Experiment	Sample ID	Sex	Age	Ki-67 proliferation index	Immunostaining positivity	Histological diagnosis	Tumor type
miRNA seq	1	M	48	5%	TTF-1, Annexin, S100, EMA, CD63	Spindle cell oncocytoma	Primary
miRNA seq	2			Normal			
miRNA seq	3	F	68	5–8%	TTF-1, Annexin, Gal-3, S100, EMA, CD63	Spindle cell oncocytoma	Primary
miRNA seq	4	M	69	< 5%	TTF-1, Annexin, Gal-3	Spindle cell oncocytoma	Primary
miRNA seq	5	M	43	1.5%	TTF-1, Annexin, Gal-3, S100, EMA	Spindle cell oncocytoma	Primary
miRNA seq	6	M	44	5%	TTF-1, Annexin, Gal-3, S100, EMA	Spindle cell oncocytoma	Recurrent 1
miRNA seq	7	M	49	4–8%	TTF-1, Annexin, Gal-3, S100, EMA	Spindle cell oncocytoma	Recurrent 2
miRNA seq	8			Normal			
miRNA seq	9	M	52	4–8%	TTF-1, Annexin, Gal-3, S100, EMA	Spindle cell oncocytoma	Recurrent 3
GSE51618							
Transcriptome data	GSM1249410	M	NA	4%	NA	Non-invasive pituitary oncocytoma	NA
Transcriptome data	GSM1249411	M	NA	1%	NA	Non-invasive pituitary oncocytoma	NA
Transcriptome data	GSM1249413	M	NA	2%	NA	Non-invasive pituitary oncocytoma	NA
Transcriptome data	GSM1249421	M	NA	Normal			
Transcriptome data	GSM1249425	M	NA	Normal			
Transcriptome data	GSM1249423	F	NA	Normal			

Additional six samples’ transcriptome data were obtained from NBCI GEO database (GSE51618) for gene expression reanalysis and for tissue specific target prediction (Table 1).

### RNA Extraction and miRNA Expression Profile by Next-Generation Sequencing

Total RNA was isolated from FFPE samples using the RecoverAll Total Nucleic Acid Isolation Kit (AM1975, Thermo Fisher Scientific, Waltham, MA, USA).

For miRNA sequencing, libraries were prepared using 5 μl RNA and the QIAseq™ miRNA Library Kit (Cat#331505, Qiagen, Hilden, Germany) following the manufacturer’s instructions. All procedures were done as previously described [18]. Briefly, following adapter ligation first at 3′ then at 5′ end of miRNAs universal reverse-transcription was performed on each sample. RT primers contained the Unique Molecular Indexes (UMIs) as well. cDNA was cleaned up using magnetic beads included in the library preparation kit. Next-generation sequencing was run on Illumina MiSeq instrument using MiSeq Reagent Kit v3 150-cycle (MS-102-3001, Illumina, San Diego, USA).

After sequencing primary data analysis was performed on FASTQ files using Qiagen GeneGlobe Data Analysis Center. Raw reads were processed by trimming off the 3′ adapter and low quality bases using cutadapt (http://cutadapt.readthedocs.io/en/stable/guide.html). Following trimming, insert sequences and UMI sequences were identified. Then sequences were aligned to miRBase v21 mature miRNA sequences using bowtie algorithm (http://bowtie-bio.sourceforge.net/index.shtml). For each sample all reads assigned to a particular miRNA ID was counted and the associated UMIs were clustered to count unique molecules. UMI read counts were used to calculate differential expression. Reads were normalized by TMM and by GeNorm [19, 20]. For further analysis miRNAs were selected if both normalization method yielded significantly different expression among groups by ANOVA combined with Fisher post hoc test.

### Analysis of Transcriptome Data

Transcriptome data of 3 pituitary oncocytoma and 3 normal pituitary tissue were obtained from NBCI GEO Database (GSE51618) measured on Agilent-014850 Whole Human Genome Microarray 4x44K G4112F platform. Data analysis was performed by Genespring GX 14.9 Software (Agilent Tech Inc, Santa Clara, CA, USA). Raw data were filtered by percentile (lower cut-off: 20). Fold change filter was set to twofold, and then unpaired t-test was used to identify significant gene expression changes (p < 0.05) with multiple testing correction (Benjamini–Hochberg) to control the false discovery rate (FDR) and to get statistically reliable results.

### Bioinformatics Analysis

Tissue specific target prediction was performed using significantly differentially expressed miRNA and mRNA lists. Target prediction was performed by TargetScan (http://www.targetscan.org/vert_72/) and miRecords (http://c1.accurascience.com/miRecords/). For further filtering miRNA-target pairs were included if opposite expressional change was detected by Ingenuity Pathway Analysis microRNA Target Filter (https://www.qiagenbioinformatics.com/products/features/microrna-target-filter/). Pathway and Gene ontology analysis were performed using Ingenuity Pathway Analysis, DAVID Bioinformatics Resources 6.8 (https://david.ncifcrf.gov/), ToPPFun (https://toppgene.cchmc.org/enrichment.jsp) and Panther Classification System (http://www.pantherdb.org/). Gene Ontology term summarizing and removal of redundant GO terms were performed by Revigo (reduce and visualize gene ontology; http://revigo.irb.hr/). For functional analysis and annotation clustering of GO terms DAVID Bioinformatics Resources 6.8 was applied. p values < 0.05 were considered significant.

### Luciferase Reporter Vector Construction

ACO2 3′-UTR (ENST00000216254.8) was amplified by PCR from genomic human DNA using the following oligonucleotides: 5′-CCATCCTCCTGAACCACACC-3′ forward and 5′-GCCTCCACTGACCTTGACTG-3′ reverse primers. The amplified sequence was cloned (5′ → 3′) into pGL3 Control vector (Promega, Madison, WI) at the 3′ end of the firefly luciferase gene at *Xba*I restriction site (pACO2). pGL3 Control vector was used as negative control.

### Transfection, Dual-Luciferase Assay, Proliferation Assay

HeLa cells were plated at 10^4^ cells per well in 96-well plates on the day before transfection. Cells were cotransfected with pACO2 [75 ng pACO2 firefly vector or negative control together with 75 ng renilla (pRL-Promega) vector] and miRNA mimics [30 nM any of miR-744-5p, miR-127-3p mirVana™ miRNA Mimics (ID: MC13027, MC10400) or with Non-targeting Control miRNA mimic #1 (4464058, Thermofisher Scientific, Grand Island, NY, USA) using PANFect A-plus Transfection Reagent (P02-8110, PAN Biotech)]. Luciferase assay was performed 24 h later using Dual-Glo luciferase assay system (E2920, Promega, Madison, WI) according to the manufacturer’s protocol. Firefly luciferase activity was adjusted for transfection efficiency by normalizing to renilla luciferase activity of each sample. For investigating cell proliferation HeLa and H295R cells were transfected with 30 nM miRNA mimics using Lipofectamine RNAiMAX (13778030, Thermofisher Scientific). After 24 h cell proliferation was measured by AlamarBlue reagent (DAL1025, Thermofisher Scientific) on Varioskan™ Flash Multimode Reader (Thermofisher Scientific).

### Immunohistochemistry

Formalin-fixed paraffin-embedded tissue sections were subjected to immunohistochemical analysis at the 1st Department of Pathology and Experimental Cancer Research, Semmelweis University. 4 µm-thick sections were cut and mounted on SuperFrost/Plus slides and stored at 4 °C until the staining. Slides were deparaffinised, rehydrated, and endogenous peroxidases were inhibited with 10% H 2 O 2 for 20 min in methanol. Antigen retrieval was achieved by incubating slides at 100 °C in TRS (10 mM Tris, 1 mM EDTA, 0.05% Tween 20, pH = 9) for 3 min. Staining was performed using the Novolink Polymer Detection System (Peroxidase/DAB+, Rabbit, Novocastra Laboratories, Newcastle, UK). Non-specific binding was blocked for 10 min at room temperature (RT) using Novocastra™ Protein Block. 0.1% Triton X-100 in TBS was applied (15 min, RT). Slides were then incubated with rabbit polyclonal anti-Drosha (ab12286, 1:100) overnight at 4 °C. Subsequently, the rabbit Novolink Polymer was applied for 30 min at RT. The primary antibody binding to tissue sections were visualized using 3,3′-diaminobenzidine (DAB, Novolink Kit, 1:20) for 4 min, and counterstained with hematoxylin.

## Results

### Global Downregulation of miRNA Expression in Pituitary Oncocytoma

**Fig. 1:**
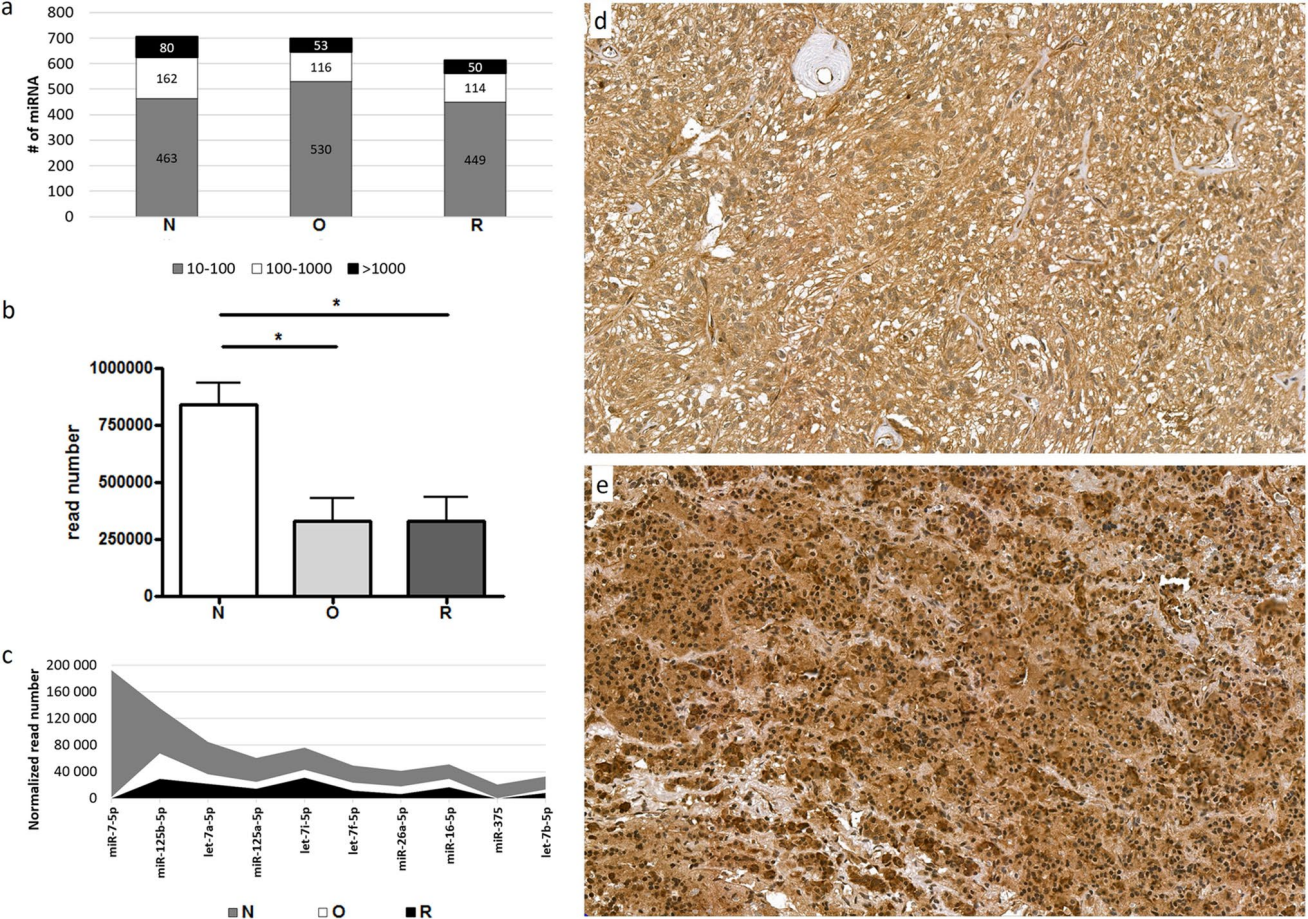
**a** miRNAs expressed in pituitary oncocytoma, recurrent oncocytoma and normal tissue in different expressional ranges. Grey represents the number of miRNA expressed in low range (between 10 and 100 normalized reads), white represents the number of miRNA expressed in middle range (between 100 and 1000 normalized reads) and black represents the number of strongly expressed miRNAs (> 1000 normalized reads). **b** Overall miRNA read numbers in normal pituitary, onocytoma and recurrent oncocytoma. *p < 0.05. **c** Ten most abundant miRNAs expression in pituitary oncocytoma (white), recurrent oncocytoma (black) and normal tissue (grey). DROSHA immunostaining in **d** Oncocytoma and **e** normal pituitary. By CaseViewer v2.1 software (3DHISTECH Ltd.) quantification strong staining appeared in 21% in oncocytoma and 41% of normal pituitary. *N* normal pituitary, *O* spindle cell onocytoma and *R* recurrent oncocytoma

**Fig. 2:**
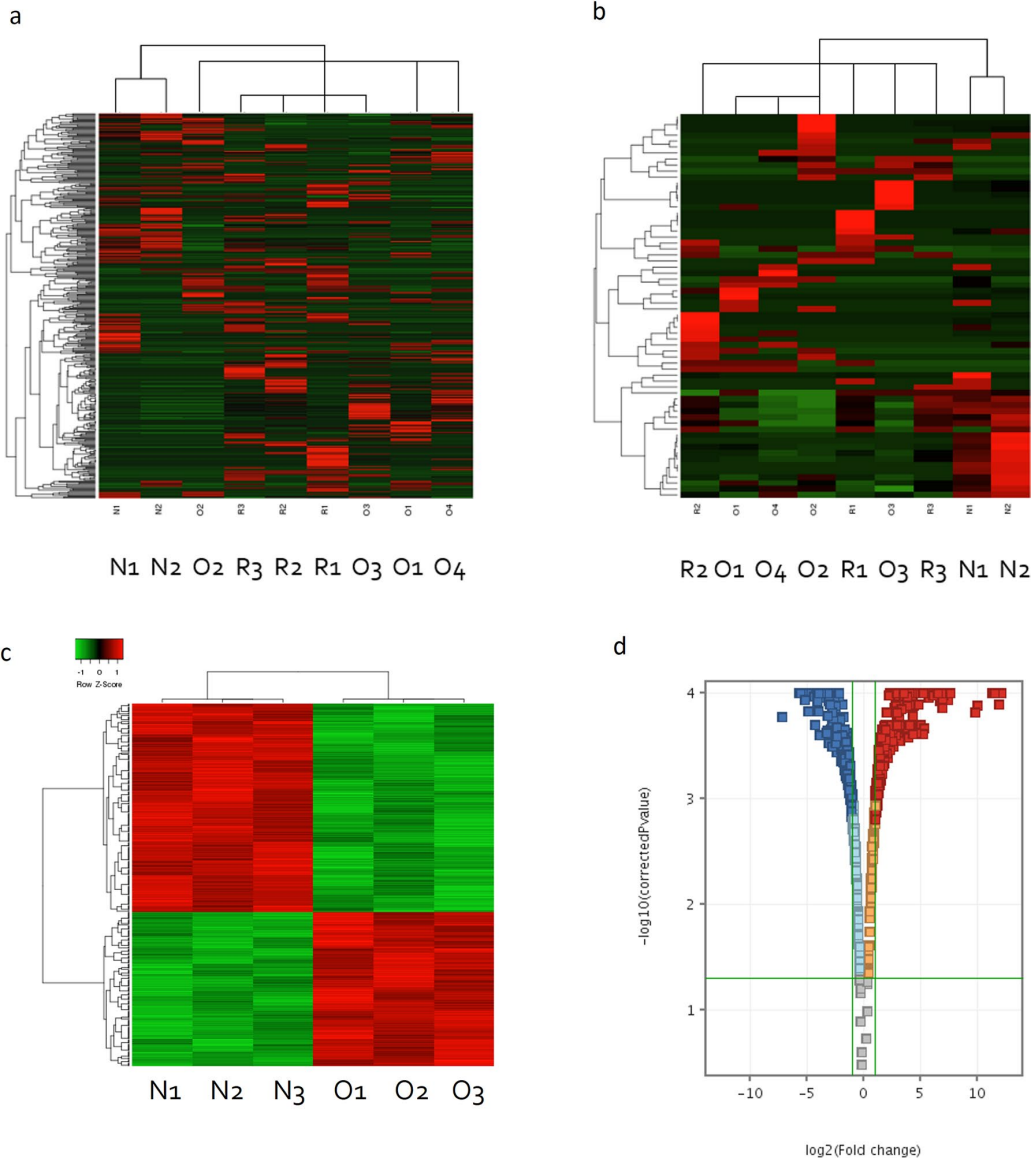
**a** Whole miRNA expression profile differentiate pituitary oncocytoma, recurrent oncocytoma and normal tissue. **b** Significant miRNAs between pituitary oncocytoma, recurrent oncocytoma and normal tissue. **c** Pituitary oncocytoma and normal pituitary samples were separated by gene expression profile. **d** Significant differentially expressed genes in pituitary oncocytoma compared to normal tissue (with a fold change cut-off:2 and p < 0.05 after FDR adjustment). *N* normal pituitary, *O* spindle cell onocytoma, *R* recurrent oncocytoma

Altogether 4,000,965 reads were generated, with an average of 889,103 reads/sample. More than half of the miRNAs were in low expression range between 10 and 100 reads. 23%, 17% and 19% of the miRNAs were between 100 and 1000 read and only 11%, 8% and 8% of the miRNAs appeared in the highest expression range (> 1000 reads) in the normal, oncocytoma and recurrent oncocytoma groups, respectively (Fig. 1a). We found global miRNA downregulation in oncocytoma vs. normal and assessing the most abundant 10 miRNAs showed lower expression in the oncocytoma and recurrent oncocytoma groups compared to the normal tissue as well (Fig. 1b, c). Investigating the mechanism behind miRNA downregulation we found lower expression of DROSHA (Drosha Ribonuclease III) on mRNA level. In a pilot experiment we detected decreased DROSHA protein level in sample where adjacent normal tissue was available (Fig. 1d, e). Additionally, 40% of downregulated miRNAs were located on chromosome 14 at 14q32 region. Whole miRNA profile was able to discriminate pituitary oncocytomas from normal tissue (Fig. 2a).

### Tissue Specific Target Prediction Using Pituitary Oncocytoma Transcriptome Data Revealed the Role of miRNAs in Metabolic Process

We identified 54 differentially expressed miRNAs in pituitary oncocytoma compared to normal tissue and 8 miRNA in recurrent oncocytomas vs. primary tumor (Fig. 2b, Online Resource 1).

By reanalysing mRNA expression data, 485 differentially expressed genes were identified (Fig. 2c, d). The most significant pathway affected by these genes was “cell cycle” (Table 2).

**Table 2:** Pathway analysis of pituitary oncocytoma transcriptome

Database					
IPA		DAVID		TOPPFUN	
Pathway	p-value	Pathway	p-value	Pathway	p-value
CELL CYCLE: G1/S	0.0025	PI3K-AKT SIGNALING PATHWAY (*Kegg Pathway*)	0.005	GROWTH HORMONE SIGNALING	0.000002
CELL CYCLE: G2/M	0.0027	CELL CYCLE: G1/S (*Biocarta Pathway*)	0.016		
GROWTH HORMONE SIGNALING	0.0027	RAP1 SIGNALING PATHWAY (*Kegg Pathway*)	0.026		
CELL CYCLE REGULATIONS	0.816	CELL CYCLE REGULATION (*Biocarta Pathway*)	0.076		

Next, we performed miRNA target prediction using TargetScan and miRecords databases. For further analysis we included miRNA-mRNA pairs only when additionally to the positive target prediction miRNA and its target showed opposite expression pattern in pituitary oncocytoma vs. normal tissue.

Pathway analysis among others showed “Tricarboxylic acid cycle (TCA cycle)” and “Cell cycle” pathways altered by miRNAs (Online Resource 2).

**Fig. 3:**
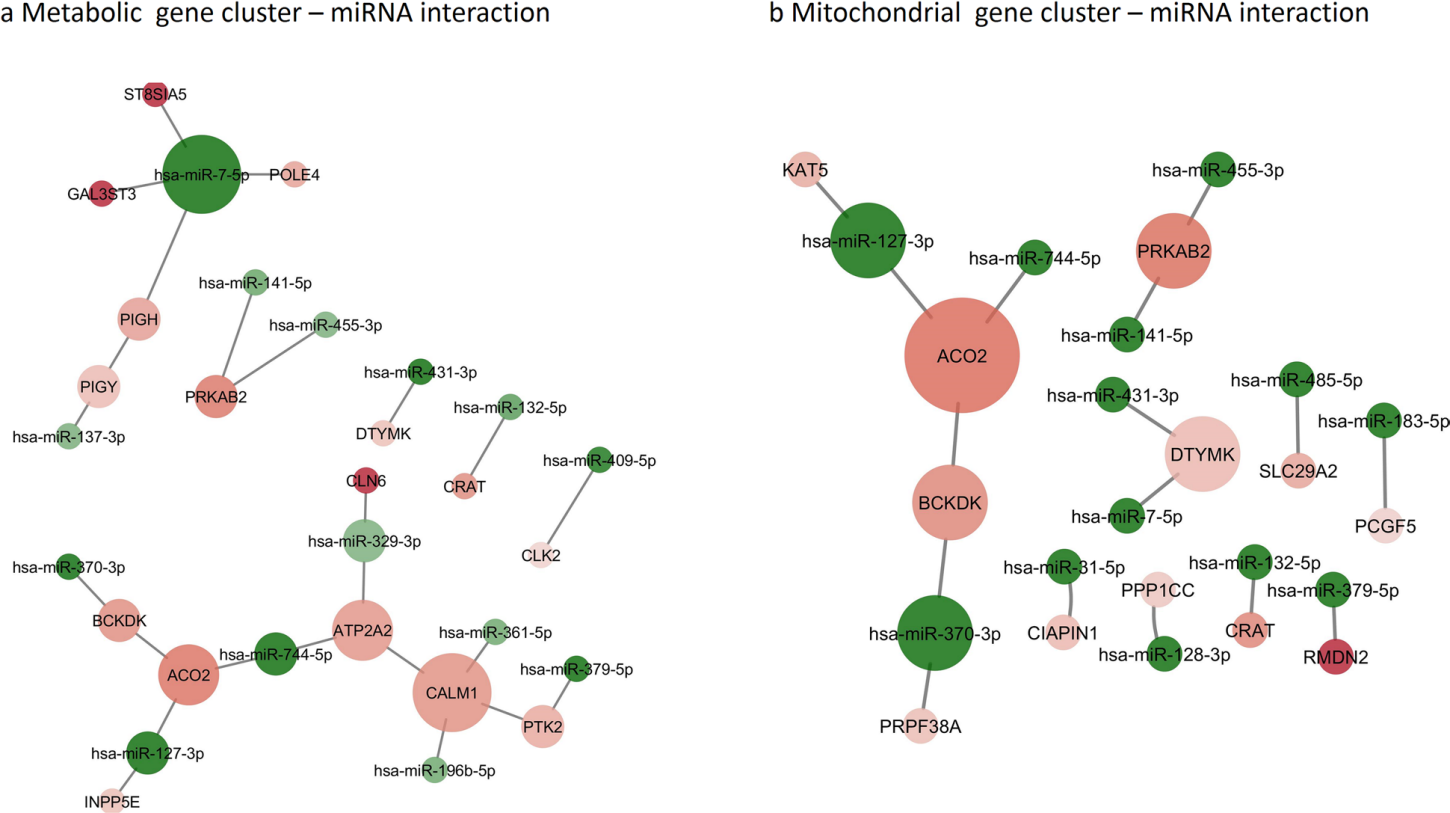
miRNA-target interaction network of oncocytoma on “Metabolic” gene cluster (**a**) and “Mitochondrial” gene cluster (**b**). Node size indicates interaction number, node color shades indicates expression (green: significant downregulation, red: significant upregulation; bright color represents higher fold change, faint color represents lower fold change) (Color figure online)

Performing Gene Ontology (GO) analysis „Cellular Process” and „Metabolic process” were the most significant biological processes influenced by miRNAs (Online Resource 3, 4). After summarizing and removing the redundant GO terms “cholesterol metabolism”, “mitochondrial transport”, “acetyltransferase activity” and “cation transmembrane transporter activity” were the most important GO terms (Online Resource 5a, b). Metabolism related Biological Processes were also enriched in miRNA regulated genes (Table 3). By functional annotation clustering we identified a Metabolism and a Mitochondria related gene cluster influenced by miRNAs (Online Resource 5c, d). Using these gene clusters we constructed a miRNA-target interaction network in order to visualize miRNAs’ role in oncocytoma metabolism and mitochondrial function (Fig. 3a, b).

**Table 3:** Metabolism related Biological Processes influenced by miRNAs differentially expressed in pituitary oncocytomas

Term	Gene count	Genes	Fold enrichment	p value
GO:0006664~glycolipid metabolic process	5	GAL3ST3, PIGY, PIGH, ST8SIA5, CLN6	13.1763967	0.0005202
GO:1903509~lipopolysaccharide metabolic process	5	GAL3ST3, PIGY, PIGH, ST8SIA5, CLN6	12.8497092	0.00057189
GO:0009247~glycolipid biosynthetic process	4	GAL3ST3, PIGY, PIGH, ST8SIA5	18.564953	0.00123325
GO:0006643~membrane lipid metabolic process	5	GAL3ST3, PIGY, PIGH, ST8SIA5, CLN6	7.62164125	0.00389079
GO:0046467~membrane lipid biosynthetic process	4	GAL3ST3, PIGY, PIGH, ST8SIA5	9.01341922	0.00942844
GO:0044262~cellular carbohydrate metabolic process	5	GAL3ST3, CLK2, INPP5E, PPP1CC, CALM1	5.73732404	0.01045816
GO:0044255~cellular lipid metabolic process	9	GAL3ST3, PTK2, PIGY, PIGH, PRKAB2, ST8SIA5, INPP5E, CRAT, CLN6	2.68328539	0.01584233
GO:0044264~cellular polysaccharide metabolic process	3	GAL3ST3, PPP1CC, CALM1	9.51927438	0.03831898
GO:0008610~lipid biosynthetic process	6	GAL3ST3, PIGY, PIGH, PRKAB2, ST8SIA5, INPP5E	2.99002849	0.046311
GO:0005976~polysaccharide metabolic process	3	GAL3ST3, PPP1CC, CALM1	8.04214559	0.05189019
GO:0044723~single-organism carbohydrate metabolic process	6	GAL3ST3, CLK2, ST8SIA5, INPP5E, PPP1CC, CALM1	2.84417344	0.05523079
GO:0006629~lipid metabolic process	9	GAL3ST3, PTK2, PIGY, PIGH, PRKAB2, ST8SIA5, INPP5E, CRAT, CLN6	2.10426065	0.05563828
GO:0019538~protein metabolic process	24	BCKDK, PIGY, L3MBTL2, PRKAB2, PKN2, NDFIP1, HMG20A, UBE2C, KAT5, PPP1CC, CAMK2N1, CCNB1, URM1, PTK2, POLE4, HIF1AN, CLK2, PIGH, ST8SIA5, USP35, FBXL14, GFRA2, CALM1, CLN6	1.36762161	0.0628948
GO:1901137~carbohydrate derivative biosynthetic process	6	GAL3ST3, PIGY, PIGH, DTYMK, ST8SIA5, CALM1	2.46796002	0.08915693
GO:0010563~negative regulation of phosphorus metabolic process	5	CCNB1, UBE2C, CAMK2N1, GFRA2, CALM1	2.85811547	0.09220633
GO:0045936~negative regulation of phosphate metabolic process	5	CCNB1, UBE2C, CAMK2N1, GFRA2, CALM1	2.85811547	0.09220633
GO:0006796~phosphate-containing compound metabolic process	15	BCKDK, PIGY, PRKAB2, DTYMK, PKN2, UBE2C, PPP1CC, CAMK2N1, CCNB1, PTK2, CLK2, PIGH, INPP5E, GFRA2, CALM1	1.51295636	0.09396383
GO:0006793~phosphorus metabolic process	15	BCKDK, PIGY, PRKAB2, DTYMK, PKN2, UBE2C, PPP1CC, CAMK2N1, CCNB1, PTK2, CLK2, PIGH, INPP5E, GFRA2, CALM1	1.51001763	0.09515533
GO:0005975~carbohydrate metabolic process	6	GAL3ST3, CLK2, ST8SIA5, INPP5E, PPP1CC, CALM1	2.39817195	0.09782965

We found that mitochondrial Aconitase 2 (ACO2) was upregulated significantly (fold change 2.78, p < 0.01) in pituitary oncocytoma compared to normal tissue on mRNA level. Additionally, miR-744-5p and miR-127-3p, two miRNAs targeting ACO2 showed significant downregulation (miR-744-5p: fold change 0.20; p = 0.01 and miR-127-3p, fold change: 0.05; p < 0.01).

### In vitro investigation of the function of miR-127-3p and miR-744-5p

**Fig. 4:**
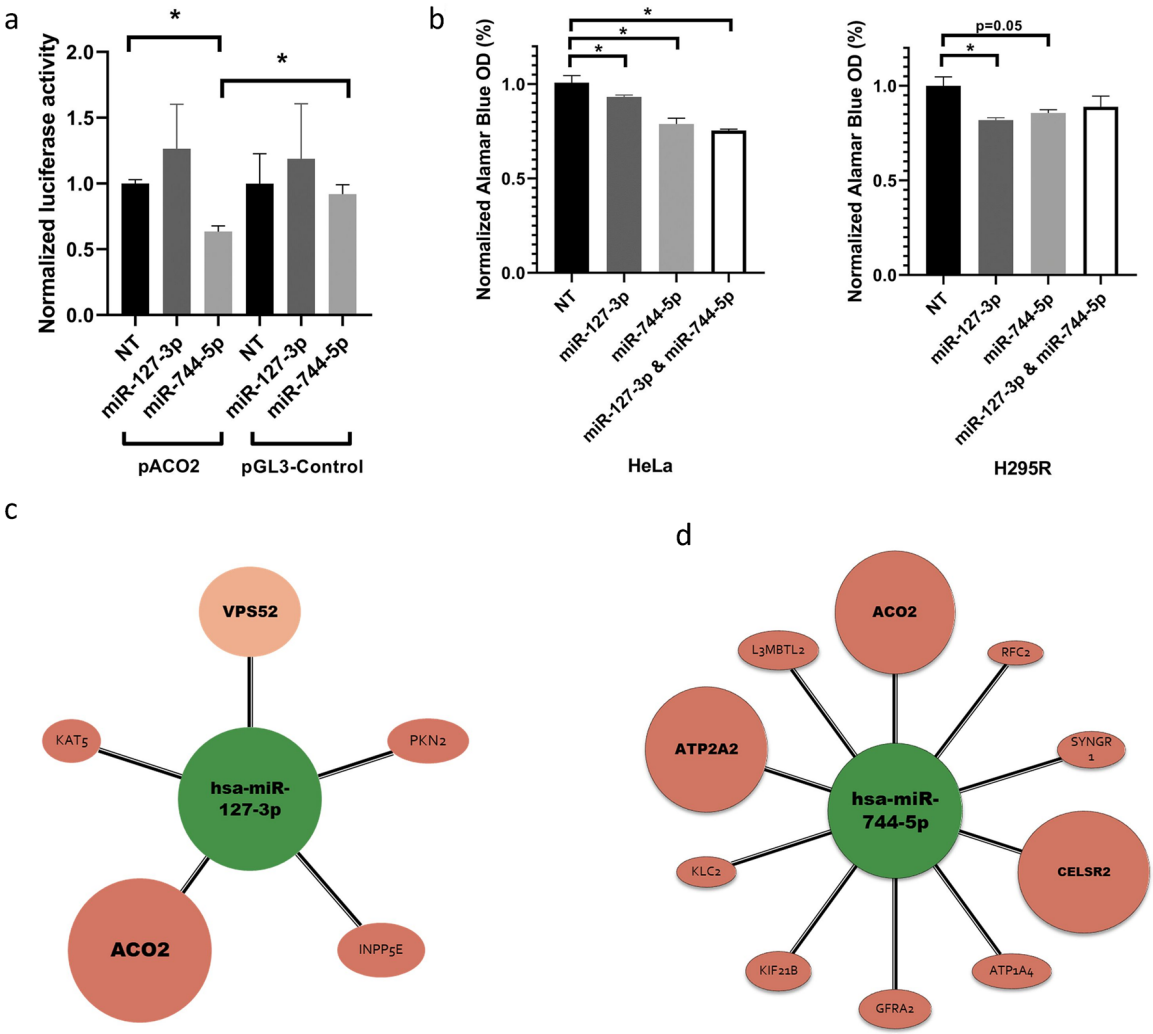
**a** Validation of Aconitase 2 (ACO2)—miRNA interaction by luciferase reporter system. Upon miR-744-5p mimics—pACO2 co-transfection relative luciferase activity decreased to 63% (p < 0.05) compared to non-targeting (NT) mimics. Additionally, miRNA mimics had no significant effect on the expression of pGL3 control vector. Normalized luciferase activity was calculated after normalizing firefly luciferase activity (pACO2) to renilla luciferase (pRL-TK) activity. Renilla vector is used to normalize between samples and to correct for transfection efficiency. **b** miRNA mimics effect on cell proliferation. Cell growth was decreased to 94%, 84%, 75% in HeLa and to 83%, 88%, 89% in H295R cells after miR-127-3p, miR-744-5p and miR-127-3p + miR-744-5p transfections, respectively. **c** miR-127-3p and **d** miR-744-5p targets based on tissue specific target prediction. Colors indicates significant gene expression alteration in oncocytoma vs. normal tissue (red: overexpression, green: underexpression) (Color figure online)

For validating miRNA-ACO2 interaction we constructed an ACO2 3′UTR firefly luciferase vector (pACO2). After co-transfection of pACO2 (with pRL-TK renilla luciferase vector for normalizing to transfection efficiency) and any of miR-127-3p, miR-744-5p or non-targeting control (NT) miRNA mimics, significantly reduced luciferase activity was detected after miR-744-5p but not after miR-127-3p transfection (Fig. 4a). For further investigation of the role of miR-127-3p and miR-744-5p HeLa and H295R cells were transfected with miRNA mimics. After 24 h posttransfection significant decrease in cell proliferation was detected (Fig. 4b).

## Discussion

MiRNA and transcriptome profiles of pituitary oncocytomas clearly separated the tumors either from normal tissue or recurrent oncocytoma samples suggesting that these expression profiles are cell type specific. Global downregulation of miRNA expression was detected in SCO and recurrent SCO vs. normal tissue. In a pilot experiment by immunohistochemistry on a slide where both oncocytoma and adjacent normal tissue was available decreased level of DROSHA was detected in oncocytoma compared to normal tissue. However, due to the low sample number, this has to be validated on en extended sample set. Additionally, by investigating the chromosomal localisation of downregulated miRNA regions, 40% of downregulated miRNAs were identified to be located on chromosome 14 at 14q32 (DLK-MEG3) locus. This region has been frequently detected as hypermethylated in tumors including pituitary adenomas [21].

Interestingly, pathway analysis of differentially expressed genes in oncocytomas revealed mainly cell cycle alterations while miRNAs rather influenced metabolic processes. Both carbohydrate (mainly tricarboxylic acid (TCA) cycle) and lipid metabolism were influenced by miRNAs. Cholesterol metabolism, mitochondrial transport, acetyltransferase activity and cation transmembrane transporter activity were the most significantly dysregulated gene processes and functions where miRNA targets were enriched. After functional annotation clustering we identified a metabolic and a mitochondrial gene cluster.

Because mitochondrial dysfunction by Complex I variants have a major role in oncocytoma pathogenesis, we suggest a central role of miRNAs-mitochondrial Aconitase 2 (ACO2) interaction in both the metabolic and the mitochondrial gene cluster. ACO2 was overexpressed in oncocytomas compared to normal tissue. ACO2 participates in the TCA cycle and the mitochondrial respiratory complexes (Complexes I, II, and III), which facilitate electron transport [22]. It catalyses the interconversion of citrate to isocitrate via cis-aconitate during the second step of the TCA cycle. When the ACO2 were partially silenced, less efficient entrance to the S phase was observed in human fibroblast cells and it resulted in significant impairment of DNA synthesis [23]. Unfortunately, a specific mechanism has not been identified which would clarify the exact role of enzymes participating in glycolysis and TCA cycle on regulation of DNA replication. It seems though, that these metabolic pathways and the control of cell cycle, particularly DNA synthesis, are linked [23]. Germline ACO2 mutations have been described in optic neuropathy with encephalopathy and cerebellar atrophy, and in infantile cerebellar-retinal degeneration [24, 25].

It has been described that hypoxia through HIF-1α upregulated ACO2 and lactate dehydrogenase A (LDHA) [26, 27]. Interestingly, in pituitary oncocytoma HIF-1α signalling pathway was not upregulated which is in line with low lactate and LDHA levels [14]. Therefore the cause of ACO2 upregulation should be different in SCO. Our results suggest that downregulated miRNAs have a role in ACO2 overexpression. In literature there was no experimentally confirmed miRNA-ACO2 interaction yet although hypoxia inducible miR-210 inhibited ACO2 activity through downregulation of biogenesis and integrity of iron–sulfur cluster assembly protein (ISCU) [28]. In this current work we validated ACO2 as a target of miR-744-5p by luciferase reporter system. Additionally, both miR-127-3p and miR-744-5p significantly decreased cell proliferation in two independent human cell lines that may suggest their “general tumor suppressor role”. Both on pACO2 activity and cell proliferation the two miRNAs moderate (~ 20%, but significant) effect refers to fine-tuning regulation, a characteristic feature of miRNA activity. We concluded that the tumor suppressor role of miR-127-3p and miR-744-5p can be mediated at least partly through regulation of ACO2.

In addition to targeting ACO2 in pituitary oncocytoma, miR-127-3p was downregulated in gonadotroph pituitary adenomas and glioblastomas too [29, 30]. It has tumor suppressor effect via inhibiting proliferation by targeting KMT5a (Histone-Lysine *N*-Methyltransferase KMT5A) and ITGA6 (Integrin, Alpha 6) [31, 32]. In pituitary oncocytoma, we found that miR-127-3p by targeting Protein Kinase 2 (PKN2) and Inositol Polyphosphate-5-Phosphatase E (INPP5E) also regulated cell proliferation [33, 34] (Fig. 4c).

Downregulation of miR-744-5p has been described in several tumors including growth hormone (GH) secreting pituitary adenoma, pancreas, ovarian and head-and-neck cancer as well [35, 36]. Its tumor suppressor role was demonstrated by targeting MAP2K4 and BCL2 [35, 37]. In pituitary oncocytoma miR-744-5p also targets ATP1A4 that is involved in carbohydrate metabolism indirectly through participating in carbohydrate digestion and absorption pathway (Fig. 4d). GFRA2 is another overexpressed target of miR-744-5p in SCO. GFRA2 stands for Glial cell line-derived neurotrophic factor (GDNF) Family Receptor Alpha 2 that is a neurotrophic factor playing key role in the control of neuron survival and differentiation.

Unfortunately, pituitary oncocytomas are extremely rare and there is no available in vitro (cell line) or in vivo (animal) model for this tumor type. Therefore it is not possible to validate the real functional effects of ACO2 overexpression and its targeting miRNAs exactly. However, mRNA and miRNA expression profiles are highly characteristic to each cell type, our data and bioinformatical associations extend the previously described metabolic alterations described in pituitary SCOs and add miRNAs as important players in this pathway.

In summary, our study is the first reporting miRNA expression profile of pituitary oncocytoma. Based on our data we propose that potentially primary changes in miRNA profile may have an important role in shaping the gene expression profile characteristic for oncocytomas. Bioinformatics analysis showed that miRNAs influence cell proliferation, carbohydrate and lipid metabolism that extends earlier reports showing downregulated TCA cycle and enhancement of pentose phosphate and phospholipid synthesis based on tissue metabolome analysis. We propose the central role of miR-744-5p targeting ACO2 in the regulation of TCA cycle in SCO. As previous reports described that Complex I mutations themselves were not sufficient for mitochondrial proliferation we concluded that differentially expressed miRNAs in pituitary oncocytoma have a supplementary role in tumorigenesis and mitochondrial dysfunction and metabolism.

## Electronic supplementary material

Below is the link to the electronic supplementary material.

Supplementary material 1 (PDF 222 kb) **Online Resource 1** Differentially expressed miRNAs in primary and recurrent spindle cell oncocytoma of the pituitary

Supplementary material 2 (PDF 98 kb) **Online Resource 2** Pathway analysis of miRNA regulated genes in pituitary oncocytoma vs. normal tissue by Panther Classification System

Supplementary material 3 (PDF 120 kb) **Online Resource 3** Gene Ontology (**a** Biological Process; **b** Molecular Function) analysis of significant differentially expressed genes influenced by differentially expressed miRNAs in oncocytoma vs. normal tissue

Supplementary material 4 (PDF 98 kb) **Online Resource 4** miRNA regulated genes associated with Cellular and Metabolic process GO terms

Supplementary material 5 (PDF 790 kb) **Online Resource 5 a** Functional analysis of differentially expressed miRNA in oncocytoma. **a** GO Biological Process representative term summary after removal of redundant terms. **b** GO Molecular Function representative term summary after removal of redundant terms. **c** Metabolic gene cluster identified by GO term Functional annotation. **d** Mitochondrial gene cluster identified by GO term Functional annotation
