# Supplementary material for: Differentially Expressed miRNAs Influence Metabolic Processes in Pituitary Oncocytoma
Source: Neurochem Res. 2019 Apr 3;44(10):2360–71. doi: 10.1007/s11064-019-02789-2 (PMC6776564; doi:10.1007/s11064-019-02789-2)
Supplement: Supplementary file 1 — Supplementary material 1 (PDF 222 kb) Online Resource 1 Differentially expressed miRNAs in primary and recurrent spindle cell oncocytoma of the pituitary [file 11064_2019_2789_MOESM1_ESM.pdf]

Online Resource 1. Differentially expressed miRNAs in primary and recurrent spindle cell oncocytoma of the pituitary. N: normal pituitary; O: oncocytoma; R: recurrent oncocytoma, BH: Benjamini-Hochberg procedure for fold discovery rate correction

**miRNA Name**	**ANOVA p value**	**Fold change (O vs. N)**	**Fisher post-hoc p value (O vs. N)**	**BH adjusted p value (O vs. N)**	**Fold change (R vs. O)**	**Fisher post-hoc p value (R vs. O)**	**BH adjusted p value (R vs. O)**
hsa-miR-379-5p	0,000	-2,824	0,000	0,000	-2,251	0,000	0,000
hsa-miR-127-3p	0,000	-2,889	0,000	0,000	-2,272	0,000	0,001
hsa-miR-154-5p	0,002	-2,347	0,001	0,004	-2,044	0,002	0,004
hsa-miR-382-5p	0,003	-2,124	0,002	0,005	-1,872	0,003	0,005
hsa-miR-369-5p	0,000	-3,578	0,000	0,000	-2,596	0,005	0,005
hsa-miR-411-5p	0,006	-1,806	0,004	0,011	-1,513	0,008	0,012
hsa-miR-494-3p	0,003	-2,258	0,001	0,003	-1,350	0,030	0,030
hsa-miR-146b-5p	0,096	-0,185	0,929	0,929	-2,094	0,046	0,137
hsa-miR-299-5p	0,002	-1,535	0,001	0,002	-0,646	0,073	0,073
hsa-miR-874-5p	0,082	-0,306	0,382	0,382	0,883	0,083	0,125
hsa-miR-136-3p	0,051	-2,069	0,022	0,065	-1,492	0,096	0,143
hsa-miR-376b-3p	0,002	-2,439	0,001	0,002	-1,089	0,099	0,099
hsa-miR-744-5p	0,001	-1,522	0,002	0,002	1,246	0,106	0,106
hsa-miR-376a-3p	0,042	-1,777	0,016	0,049	-1,129	0,117	0,161
hsa-miR-455-3p	0,079	2,569	0,035	0,104	0,909	0,134	0,201
hsa-miR-551b-3p	0,002	-1,793	0,002	0,003	1,495	0,167	0,167
hsa-miR-485-3p	0,006	-1,714	0,002	0,006	-0,669	0,175	0,175
hsa-miR-654-3p	0,004	-3,323	0,002	0,005	-1,532	0,240	0,240
hsa-miR-769-3p	0,090	-0,577	0,138	0,206	0,579	0,240	0,240
hsa-miR-598-3p	0,121	-0,900	0,155	0,233	1,067	0,309	0,309
hsa-miR-381-3p	0,004	-3,065	0,001	0,004	-1,204	0,309	0,309
hsa-miR-409-3p	0,002	-3,010	0,001	0,003	-1,062	0,327	0,327
hsa-miR-574-3p	0,024	-1,147	0,021	0,032	0,580	0,375	0,375
hsa-miR-129-2-3p	0,042	-1,869	0,034	0,051	1,472	0,482	0,482
hsa-miR-95-3p	0,069	-0,549	0,056	0,085	0,197	0,489	0,489
hsa-miR-370-3p	0,005	-2,257	0,002	0,005	-0,544	0,494	0,494
hsa-miR-129-1-3p	0,112	-2,032	0,045	0,136	-0,768	0,539	0,539
hsa-miR-485-5p	0,001	-2,569	0,001	0,001	-0,422	0,616	0,616
hsa-miR-129-5p	0,002	-3,538	0,001	0,001	2,252	0,630	0,630
hsa-miR-487b-3p	0,028	-3,893	0,012	0,034	-1,292	0,687	0,687
hsa-miR-431-5p	0,083	-2,182	0,053	0,080	1,058	0,704	0,704
hsa-miR-212-5p	0,090	-2,070	0,058	0,087	0,938	0,705	0,705
hsa-miR-1180-3p	0,092	-1,115	0,059	0,088	0,264	0,718	0,718
hsa-miR-148b-3p	0,086	-0,640	0,054	0,081	0,115	0,723	0,723
hsa-miR-409-5p	0,006	-2,305	0,003	0,007	-0,315	0,745	0,745
hsa-miR-141-5p	0,037	-2,526	0,022	0,032	0,839	0,757	0,757

hsa-miR-7-2-3p	0,002	-2,970	0,001	0,002	0,515	0,775	0,775
hsa-miR-31-5p	0,019	-1,607	0,011	0,016	0,230	0,776	0,776
hsa-miR-212-3p	0,008	-3,644	0,004	0,007	1,621	0,778	0,778
hsa-miR-137	0,056	-3,060	0,033	0,050	1,632	0,783	0,783
hsa-miR-323a-3p	0,009	-4,115	0,004	0,010	-0,922	0,789	0,789
hsa-miR-329-3p	0,080	-2,304	0,036	0,086	-0,407	0,809	0,809
hsa-miR-200b-5p	0,016	-1,695	0,007	0,018	-0,163	0,818	0,818
hsa-miR-889-3p	0,059	-2,587	0,033	0,049	0,589	0,841	0,841
hsa-miR-539-3p	0,011	-1,997	0,005	0,012	-0,161	0,854	0,854
hsa-miR-132-5p	0,026	-3,986	0,014	0,022	1,437	0,874	0,874
hsa-miR-7-5p	0,000	-5,872	0,000	0,000	2,156	0,892	0,892
hsa-miR-376c-3p	0,102	-2,207	0,048	0,101	-0,233	0,897	0,897
hsa-miR-182-5p	0,002	-4,557	0,001	0,002	0,682	0,911	0,911
hsa-miR-361-5p	0,048	-0,924	0,025	0,041	0,045	0,913	0,913
hsa-miR-431-3p	0,000	-2,897	0,000	0,000	0,066	0,921	0,921
hsa-miR-183-5p	0,001	-5,473	0,000	0,001	1,092	0,926	0,926
hsa-miR-200a-5p	0,002	-4,864	0,001	0,002	-0,292	0,960	0,960
hsa-miR-375	0,016	-5,252	0,008	0,015	0,717	0,964	0,964
hsa-miR-96-5p	0,009	-4,551	0,004	0,009	-0,237	0,969	0,969
hsa-miR-200a-3p	0,008	-5,137	0,004	0,008	0,431	0,971	0,971
hsa-miR-141-3p	0,009	-5,022	0,004	0,008	0,354	0,974	0,974
hsa-miR-200c-3p	0,006	-5,900	0,003	0,006	0,639	0,975	0,975
hsa-miR-429	0,015	-5,460	0,007	0,013	0,531	0,975	0,975
hsa-miR-132-3p	0,014	-4,408	0,007	0,013	0,193	0,978	0,978
hsa-miR-196b-5p	0,049	2,110	0,025	0,044	-0,008	0,982	0,982
hsa-miR-200b-3p	0,005	-5,351	0,003	0,005	0,048	0,996	0,996
hsa-miR-323b-3p	0,005	-3,680	0,002	0,005	0,013	0,997	0,997
hsa-miR-128-3p	0,016	-1,468	0,008	0,015	0,001	0,999	0,999
