# Supplementary material for: Differentially Expressed miRNAs Influence Metabolic Processes in Pituitary Oncocytoma
Source: Neurochem Res. 2019 Apr 3;44(10):2360–71. doi: 10.1007/s11064-019-02789-2 (PMC6776564; doi:10.1007/s11064-019-02789-2)
Supplement: Supplementary file 2 — Supplementary material 2 (PDF 98 kb) Online Resource 2 Pathway analysis of miRNA regulated genes in pituitary oncocytoma vs. normal tissue by Panther Classification System [file 11064_2019_2789_MOESM2_ESM.pdf]

Online Resource 2 Pathway analysis of miRNA regulated genes in pituitary oncocytoma vs. normal tissue by Panther Classification System

**Pathway accession**	**Pathway Name**	**Components**	**Subfamilies**	**Associated sequence**
P00060	Ubiquitin proteasome pathway	6	107	570
P06664	Gonadotropin releasing hormone receptor pathway	216	233	235
P00003	Alzheimer disease-amyloid secretase pathway	31	113	545
P00034	Integrin signalling pathway	46	258	1312
P00013	Cell cycle	12	31	148
P00051	TCA cycle	10	24	80
P02739	De novo pyrimidine deoxyribonucleotide biosynthesis	10	33	253
P04397	p53 pathway by glucose deprivation	13	28	184
P05912	Dopamine receptor mediated signaling pathway	27	83	428
P00004	Alzheimer disease-presenilin pathway	70	186	1056
P00053	T cell activation	45	98	564
P06587	Nicotine pharmacodynamics pathway	27	40	163
P00047	PDGF signaling pathway	36	207	1043
P00059	p53 pathway	70	104	607
P00005	Angiogenesis	77	245	1272
P00057	Wnt signaling pathway	49	464	2334
P00017	DNA replication	18	49	271
P00028	Heterotrimeric G-protein signaling pathway-rod outer segment phototransduction	15	51	313
P00056	VEGF signaling pathway	25	96	495
P00012	Cadherin signaling pathway	16	223	1074
P00010	B cell activation	37	78	430
P00026	Heterotrimeric G-protein signaling pathway-Gi alpha and Gs alpha mediated pathway	27	201	1130
P06959	CCKR signaling map	290	171	171
P00042	Muscarinic acetylcholine receptor 1 and 3 signaling pathway	12	97	515
