# Supplementary material for: Differentially Expressed miRNAs Influence Metabolic Processes in Pituitary Oncocytoma
Source: Neurochem Res. 2019 Apr 3;44(10):2360–71. doi: 10.1007/s11064-019-02789-2 (PMC6776564; doi:10.1007/s11064-019-02789-2)

PANTHER GO-Slim Biological Process

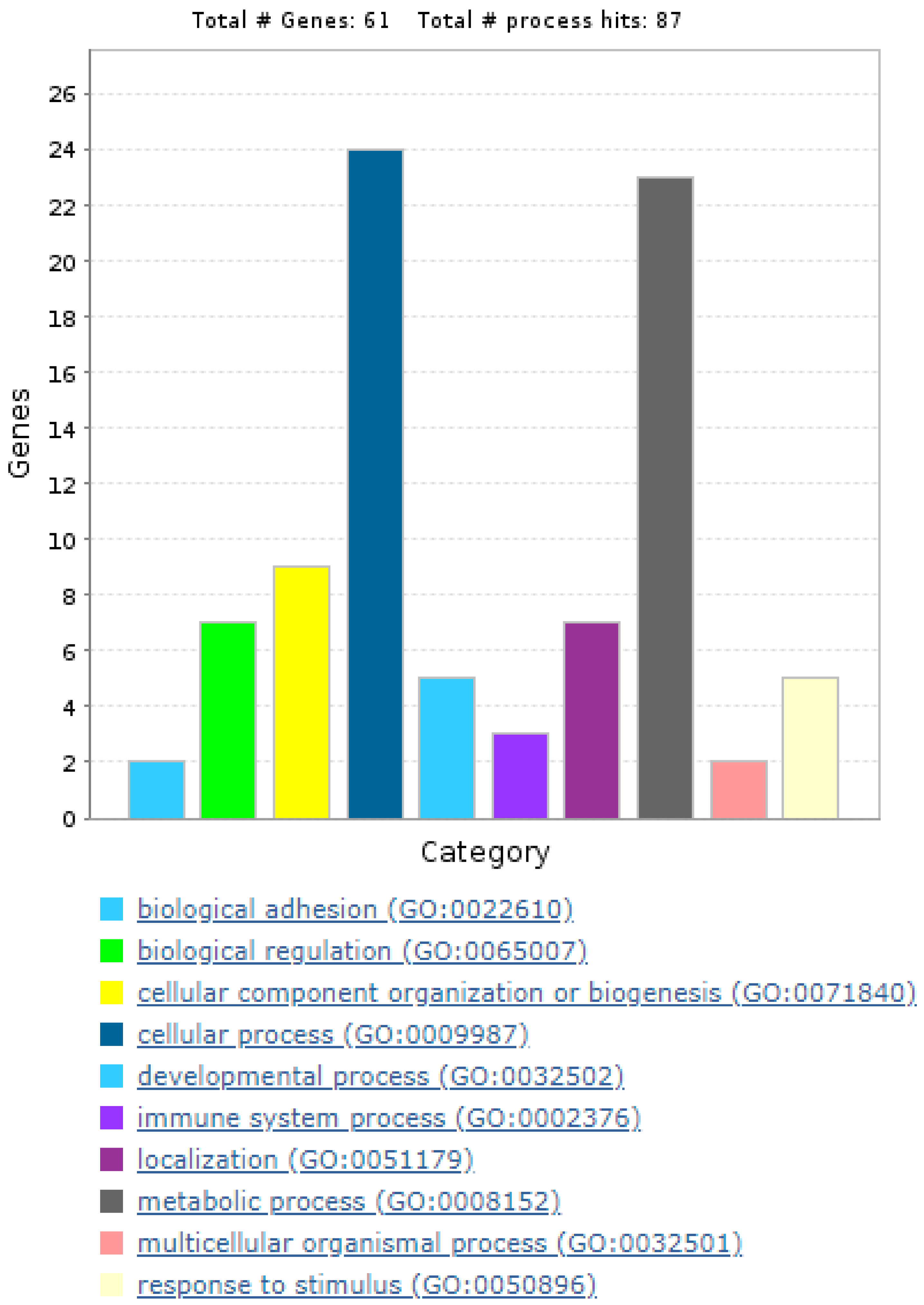

**Chart tooltips are read as: Category name (Accession): # genes; Percent of gene hit against total # genes; Percent of gene hit against total # Function hits

PANTHER GO-Slim Molecular Function

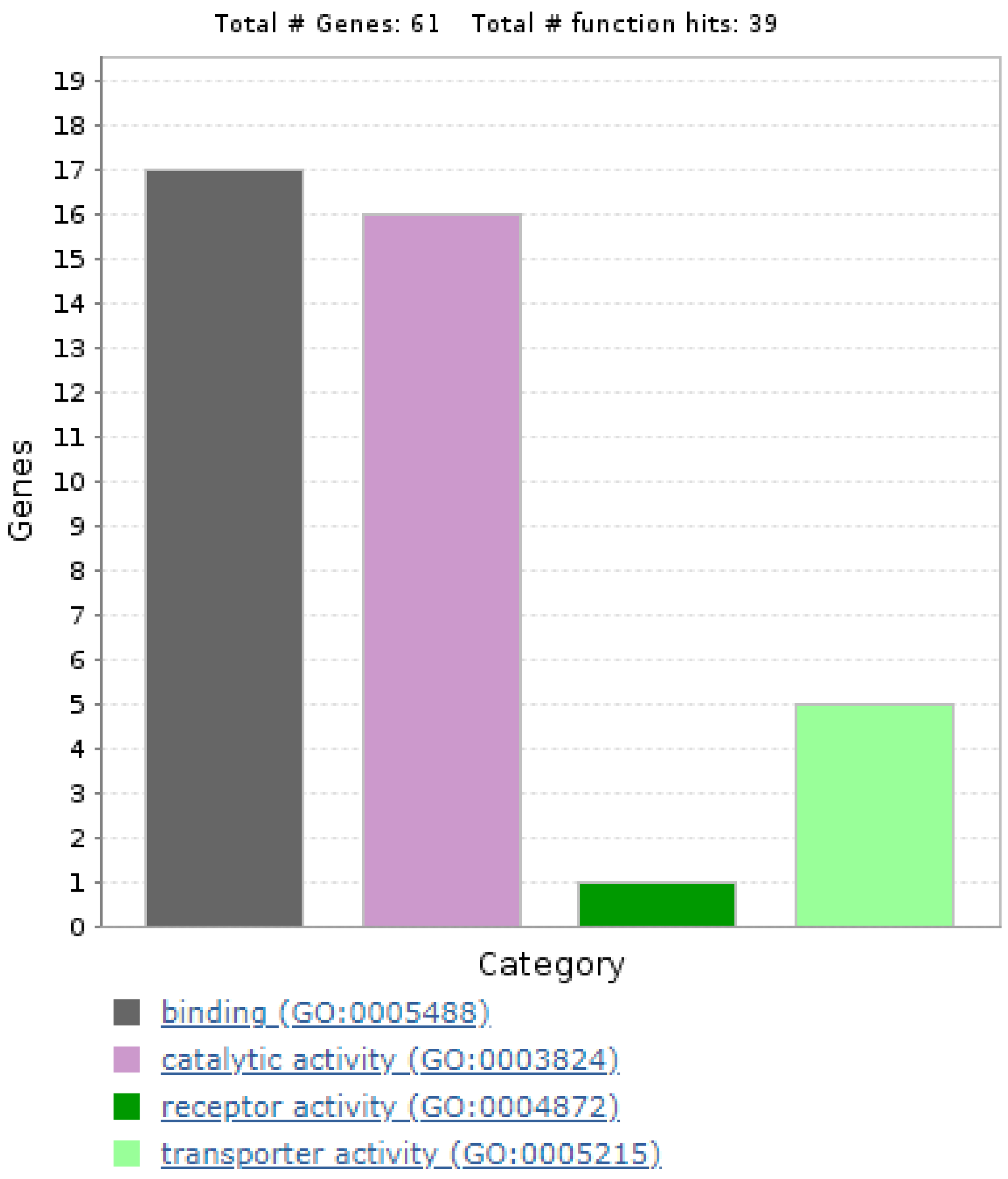

Supplement: Supplementary file 3 — Supplementary material 3 (PDF 120 kb) Online Resource 3 Gene Ontology (a Biological Process; b Molecular Function) analysis of significant differentially expressed genes influenced by differentially expressed miRNAs in oncocytoma vs. normal tissue [file 11064_2019_2789_MOESM3_ESM.pdf]
