# Supplementary material for: Differentially Expressed miRNAs Influence Metabolic Processes in Pituitary Oncocytoma
Source: Neurochem Res. 2019 Apr 3;44(10):2360–71. doi: 10.1007/s11064-019-02789-2 (PMC6776564; doi:10.1007/s11064-019-02789-2)
Supplement: Supplementary file 4 — Supplementary material 4 (PDF 98 kb) Online Resource 4 miRNA regulated genes associated with Cellular and Metabolic process GO terms [file 11064_2019_2789_MOESM4_ESM.pdf]

Online Resource 4 miRNA regulated genes associated with Cellular and Metabolic process GO terms

Cellular process		Metabolic process	
Symbol	Gene name	Symbol	Gene name
TBC1D2B	TBC1 domain family member 2B;TBC1D2B;ortholog	TBC1D2B	TBC1 domain family member 2B;TBC1D2B;ortholog
VPS52	Vacuolar protein sorting-associated protein 52 homolog;VPS52;ortholog	PPP1CC	Serine/threonine-protein phosphatase PP1-gamma catalytic subunit;PPP1CC;ortholog
PPP1CC	Serine/threonine-protein phosphatase PP1-gamma catalytic subunit;PPP1CC;ortholog	KAT5	Histone acetyltransferase KAT5;KAT5;ortholog
RILPL1	RILP-like protein 1;RILPL1;ortholog	RERE	Arginine-glutamic acid dipeptide repeats protein;RERE;ortholog
KAT5	Histone acetyltransferase KAT5;KAT5;ortholog	DTYMK	Thymidylate kinase;DTYMK;ortholog
RERE	Arginine-glutamic acid dipeptide repeats protein;RERE;ortholog	ATP2A2	Sarcoplasmic/endoplasmic reticulum calcium ATPase 2;ATP2A2;ortholog
DTYMK	Thymidylate kinase;DTYMK;ortholog	L3MBTL2	Lethal(3)malignant brain tumor-like protein 2;L3MBTL2;ortholog
ATP2A2	Sarcoplasmic/endoplasmic reticulum calcium ATPase 2;ATP2A2;ortholog	PRKAB2	5'-AMP-activated protein kinase subunit beta-2;PRKAB2;ortholog
L3MBTL2	Lethal(3)malignant brain tumor-like protein 2;L3MBTL2;ortholog	PMPCA	Mitochondrial-processing peptidase subunit alpha;PMPCA;ortholog
INPP5E	Mitochondrial-processing peptidase subunit alpha;PMPCA;ortholog	KIF21B	Kinesin-like protein KIF21B;KIF21B;ortholog
KIF21B	Kinesin-like protein KIF21B;KIF21B;ortholog	RFC2	Replication factor C subunit 2;RFC2;ortholog
RFC2	Replication factor C subunit 2;RFC2;ortholog	CCNB1	G2/mitotic-specific cyclin-B1;CCNB1;ortholog
CCNB1	G2/mitotic-specific cyclin-B1;CCNB1;ortholog	CRAT	Carnitine O-acetyltransferase;CRAT;ortholog
PKN2	Serine/threonine-protein kinase N2;PKN2;ortholog	POLE4	DNA polymerase epsilon subunit 4;POLE4;ortholog
CALM1	Calmodulin;CALM1;ortholog	PKN2	Serine/threonine-protein kinase N2;PKN2;ortholog
PTK2	Focal adhesion kinase 1;PTK2;ortholog	ATP1A4	Sodium/potassium-transporting ATPase subunit alpha-4;ATP1A4;ortholog
ATP1A4	Sodium/potassium-transporting ATPase subunit alpha-4;ATP1A4;ortholog	BCKDK	[3-methyl-2-oxobutanoate dehydrogenase [lipoamide]] kinase, mitochondrial;BCKDK;ortholog
BCKDK	[3-methyl-2-oxobutanoate dehydrogenase [lipoamide]] kinase, mitochondrial;BCKDK;ortholog	ACO2	Aconitate hydratase, mitochondrial;ACO2;ortholog
ACO2	Aconitate hydratase, mitochondrial;ACO2;ortholog		
