# Supplementary material for: Differentially Expressed miRNAs Influence Metabolic Processes in Pituitary Oncocytoma
Source: Neurochem Res. 2019 Apr 3;44(10):2360–71. doi: 10.1007/s11064-019-02789-2 (PMC6776564; doi:10.1007/s11064-019-02789-2)
Supplement: Supplementary file 5 — Supplementary material 5 (PDF 790 kb) Online Resource 5 a Functional analysis of differentially expressed miRNA in oncocytoma. a GO Biological Process representative term summary after removal of redundant terms. b GO Molecular Function representative term summary after removal of redundant terms. c Metabolic gene cluster identified by GO term Functional annotation. d Mitochondrial gene cluster identified by GO term Functional annotation [file 11064_2019_2789_MOESM5_ESM.pdf]

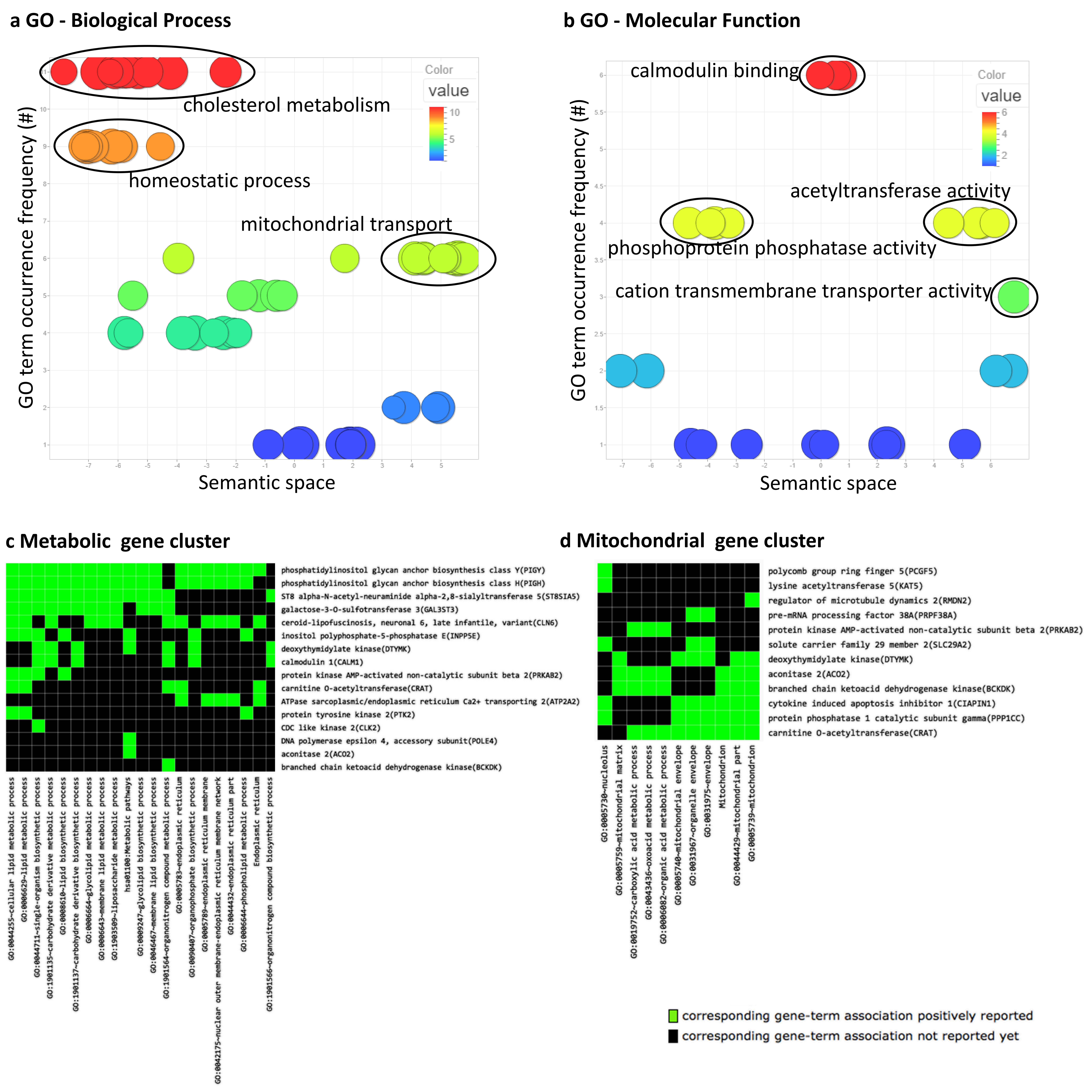
a GO - Biological Process
cholesterol metabolism
homeostatic process
mitochondrial transport
GO term occurrence frequency (#)
Semantic space
Color
value
b GO - Molecular Function
calmodulin binding
acetyltransferase activity
phosphoprotein phosphatase activity
cation transmembrane transporter activity
GO term occurrence frequency (#)
Semantic space
Color
value
c Metabolic gene cluster
phosphatidylinositol glycan anchor biosynthesis class Y(PIGY)
phosphatidylinositol glycan anchor biosynthesis class H(PIGH)
ST8 alpha-N-acetyl-neuraminide alpha-2,8-sialyltransferase 5(ST8SIA5)
galactose-3-O-sulfotransferase 3(GAL3ST3)
ceroid-lipofuscinosis, neuronal 6, late infantile, variant(CLN6)
inositol polyphosphate-5-phosphatase E(INPP5E)
deoxythymidylate kinase(DTYMK)
calmodulin 1(CALM1)
protein kinase AMP-activated non-catalytic subunit beta 2(PRKAB2)
carnitine O-acetyltransferase(CRAT)
ATPase sarcoplasmic/endoplasmic reticulum Ca2+ transporting 2(ATP2A2)
protein tyrosine kinase 2(PTK2)
CDC like kinase 2(CLK2)
DNA polymerase epsilon 4, accessory subunit(POLE4)
aconitase 2(ACO2)
branched chain ketoacid dehydrogenase kinase(BCKDK)
GO:0044255~cellular lipid metabolic process
GO:0006629~lipid metabolic process
GO:0044711~single-organism biosynthetic process
GO:1901135~carbohydrate derivative metabolic process
GO:0008610~lipid biosynthetic process
GO:1901137~carbohydrate derivative biosynthetic process
GO:0006664~glycolipid metabolic process
GO:0006643~membrane lipid metabolic process
GO:1903509~liposaccharide metabolic process
hsa01100:Metabolic pathways
GO:0009247~glycolipid biosynthetic process
GO:0046467~membrane lipid biosynthetic process
GO:1901564~organonitrogen compound metabolic process
GO:0005783~endoplasmic reticulum
GO:0090407~organophosphate biosynthetic process
GO:0005789~endoplasmic reticulum membrane
GO:0042175~nuclear outer membrane-endoplasmic reticulum membrane network
GO:0044432~endoplasmic reticulum part
GO:0006644~phospholipid metabolic process
Endoplasmic reticulum
GO:1901566~organonitrogen compound biosynthetic process
d Mitochondrial gene cluster
polycomb group ring finger 5(PCGF5)
lysine acetyltransferase 5(KAT5)
regulator of microtubule dynamics 2(RMDN2)
pre-mRNA processing factor 38A(PRPF38A)
protein kinase AMP-activated non-catalytic subunit beta 2(PRKAB2)
solute carrier family 29 member 2(SLC29A2)
deoxythymidylate kinase(DTYMK)
aconitase 2(ACO2)
branched chain ketoacid dehydrogenase kinase(BCKDK)
cytokine induced apoptosis inhibitor 1(CIAPIN1)
protein phosphatase 1 catalytic subunit gamma(PPP1CC)
carnitine O-acetyltransferase(CRAT)
GO:0005730~nucleolus
GO:0005759~mitochondrial matrix
GO:0019752~carboxylic acid metabolic process
GO:0043436~oxoacid metabolic process
GO:0006082~organic acid metabolic process
GO:0005740~mitochondrial envelope
GO:0031967~organelle envelope
GO:0031975~envelope
Mitochondrion
GO:0044429~mitochondrial part
GO:0005739~mitochondrion
corresponding gene-term association positively reported
corresponding gene-term association not reported yet
